# The Critical Social Processes for Standardising the Ecological Monitoring of Australian Landscapes

**DOI:** 10.1007/s00267-024-02049-2

**Published:** 2024-09-26

**Authors:** Hitje-Aikaterini Grypma, Douglas K. Bardsley, Ben Sparrow

**Affiliations:** 1 TERN Ecosystem Surveillance, School of Biological Sciences, Faculty of Science, Engineering and Technology, University of Adelaide, Adelaide, SA Australia; 2 Geography, Environment and Population, School of Social Sciences, Faculty of Arts, Business, Law and Economics, University of Adelaide, Adelaide, SA Australia

**Keywords:** Ecological monitoring, Standardisation, Natural resource management, Reflexivity, Adaptiveco-management, Australia

## Abstract

For a long time, ecological monitoring across Australia has utilised a wide variety of different methodologies resulting in data that is difficult to analyse across place or time. In response to these limitations, a new systematic approach to ecological monitoring has been developed in collaboration between the Terrestrial Ecosystem Research Network and the Australian Department of Climate Change, Energy, the Environment and Water - the Ecological Monitoring System Australia (EMSA). A qualitative approach involving focus groups and semi-structured interviews was undertaken to review perceptions of the introduction of the EMSA protocols amongst Natural Resource Management practitioners and other key stakeholders. We found that environmental management stakeholders recognise there will be many advantages from the standardisation of ecological monitoring. However, key concerns emerged regarding the capacity needed to implement the standard protocols, the utility of the resultant data for regional projects, and the scope for adaptive co-management under the EMSA. Stakeholders emphasised the need for autonomy and flexibility, so their participation in protocol development can facilitate regional adoption of the standards. Respondents’ concerns about a perceived lack of genuine consultation and acknowledgement of feedback revealed the importance of clear communication at all stages of an environmental management project aiming to standardise practices. Our findings indicate that reflexivity will be vital to address the complexity involved in standardisation of ecological monitoring. Formal processes of social learning will need to be integrated into environmental management approaches to account for the increasing complexity of socio-ecological systems as they are challenged by global change.

## Introduction

The social aspects of ecological monitoring are often overlooked. Ecological monitoring provides vital information for decision-makers and natural resource management (NRM) practitioners to make informed environmental management decisions. Successful monitoring measures ecological change and informs a range of decisions, actions and research relevant to environmental management policy and practice (Burns et al. 2018). To that end, many useful monitoring methods quantify current ecological condition in a manner which enables the forecasting of ecosystem change to improve risk management and sustainable economic and social development (Lindenmayer et al. 2015; Kühl et al. 2020). Such monitoring continues to be funded and conducted, and the resultant data interpreted and utilised by stakeholders whose varying interests and objectives inevitably influence their work in different ways. Nonetheless, the social processes involved in ecological monitoring are under-represented in the literature and are examined here in relation to the development of a systematic approach to ecological monitoring in Australia.

In this article, we initially introduce the current state and socio-cultural aspects of ecological monitoring in Australia. Subsequently, Australia’s new systematic ecological monitoring system is introduced. We use stakeholder perceptions to develop an argument for standardising ecological monitoring in Australia and in doing so, discuss the importance of the way that people conceptualise, design and enact ecological monitoring. Finally, we discuss the potential for generating mutual socio-ecological understanding for the success of future monitoring programmes.

### Socio-cultural Aspects of Ecological Monitoring

Like all social processes, attitudes of and approaches towards ecological monitoring differ between individuals and groups. As Norgaard (1992, p. 95) noted, “Environmental science as a social process entails scientists entering into discourse, debating and rethinking each other’s understandings, and approaching an interpretive consensus.” Normative environmental science alone is failing to generate simple, universally agreed solutions to complex problems. The alternative is to acknowledge that the perceptions and behaviours of key research and management actors must be understood to generate knowledge to effectively implement environmental management approaches driven by science (Wittmayer and Schäpke 2014). Without such social knowledge to inform some level of ecological monitoring consensus, conflicts materialise and perpetuate that could prevent the effective application of a systematic approach such as the Ecological Monitoring System Australia (EMSA), the focus of this research, introduced below in section “The Ecological Monitoring System Australia”.

Several key concepts have been developed in theory and practice in recognition of the social aspects of ecological monitoring. The concept of adaptive co-management acknowledges the importance of social networks and norms between stakeholders for improved collaboration to manage systems to respond to disturbances and adapt to change (Armitage 2005; Cundill and Fabricius 2009; Bardsley et al. 2021). Adaptive co-management emphasises learning-by-doing through monitoring and action (Walters and Holling 1990; Daniels and Walker 2001), with a focus on collaborative and inclusive decision-making (Borrini-Feyerabend 1996; Carlsson and Berkes 2005). Reflexivity refers to an individual or group’s ability to examine themselves in relation to their actions and interactions with others (Pienkowski et al. 2023). Social learning has been defined as a process of learning through collective, iterative reflection (Keen et al., 2005; Fernandez-Gimenez et al., 2019), or a learning outcome, such as a change in conceptions or attitudes, which becomes social learning when it is diffused through a larger network via social interaction (Reed et al. 2010). The emerging practice of holistic ecological monitoring is tied to a growing awareness of the importance of better knowledge of environmental conditions, as well as social and economic prosperity, to advance sustainable development (Jaegar et al. 2008; Ferrario et al. 2022). Holistic environmental monitoring aims to develop a comprehensive representation of a landscape integrated in its wider ecological context (Ferrario et al. 2022). Without knowledge of the social processes that will enable change in monitoring, environmental management stakeholders could struggle to understand change within a nation’s ecological systems or develop the adaptive capacity to manage future ecosystems.

### Ecological Monitoring in Australia

For a long time, ecological monitoring across Australia has utilised a range of ecological monitoring methodologies, resulting in data that is difficult to analyse across place, time or system (Likens and Lindenmayer 2018; Sparrow et al. 2020a; Sparrow et al. 2020b). At a local level, standardised methods can assist NRM project managers to report consistently on outcomes from their interventions. Beyond such important local goals, consistent data at a national scale enables governments and private interests to better report on the status and trajectory of a wide variety of environmental parameters. Such data are lacking in Australia, with NRM projects and practitioners often operating independently using methods that are not always comparable or sufficient to inform good environmental science or decision-making (Field et al. 2007; van Dijk et al. 2014; Scheele et al. 2019). Without the ability to compare data seamlessly across jurisdictions, important reviews such as State of the Environment reporting (Department of Climate Change, Energy, Environment and Water DCCEEW (2023a)), the Threatened Species Action Plan (DCCEEW 2023b) or Australia’s reporting on greenhouse gas emissions (DCCEEW 2023c) lack detail or accuracy. All of these actions would be enhanced by effective systematic ecological monitoring and deposition of that data in the Australian Government’s Biodiversity Data Repository (BDR). Alongside such immediate opportunities, the contemporary need for improved ecological monitoring is also driven by a requirement for more effective knowledge of approaches for sustainable development in an era of rapid environmental change. Ecological risk is generating new data demands for environmental management that will need readily available high-quality data to understand and apply across changing landscapes (Bardsley and Knierim 2020; Leclère et al. 2020).

The world is experiencing increasing rates of climate change and biodiversity loss (IPBES 2019; IPCC 2022). As society is directly implicated in generating the global ecological crisis, the conservation community is interested in working with communities to implement adaptive management and evidence-based conservation approaches (Bennett 2016; Schmidt-Traub et al., 2017). In Australia, new commercial and policy frameworks are being developed in response, such as the Nature Positive Plan (DCCEEW 2022), the Nature Repair Market (DCCEEW 2024a), and the Agricultural Sustainability Framework (McRobert et al., 2023). Such planning is being undertaken in association with opportunities for establishing national environmental standards, conservation planning, and carbon sequestration, to allow landholders to engage holistically in regenerative landscape management (Laine et al. 2021; O’Donoghue et al. 2022; DCCEEW 2024a). While it is envisioned that EMSA will primarily be used for federal government-funded NRM initiatives, broader use of the system by businesses, non-government organisations (NGOs) and other groups conducting terrestrial monitoring and research in Australia would generate a more comprehensive national ecological dataset. Together, the new approach to monitoring has the potential to guide improved monitoring and assessment across natural and anthropogenic landscapes for sophisticated ecological analysis that allows for detailed management outcomes that are unique to place and system (Cleverly et al. 2019; Zurell et al. 2020, Perry et al. 2023).

### The Ecological Monitoring System Australia

In response to the limitations described in section 1.2., a new systematic approach to ecological monitoring has been developed as a collaboration between the Terrestrial Ecosystem Research Network (TERN) and the Australian Federal Department of Climate Change, Energy, the Environment and Water (DCCEEW) and has been called the Ecological Monitoring System Australia (EMSA) (See Box 1).

Since 2021, TERN has partnered with DCCEEW to design and standardise ecological monitoring and data systems for improved decision-making related to National Landcare Programme (NLP) investments into NRM (O’Neill et al. 2020a; O’Neill et al. 2020b; DCCEEW 2024b). The three-year project developed standardised field survey protocols to assist professionals such as NRM staff, managers and environmental consultants, as well as community volunteers to collect consistent and comparable monitoring data from different sites and ecosystems. Additionally, TERN and DCCEEW developed a data collection app, an associated data exchange system and data management standards. The data entered through the app will be uploaded to the Australian Government’s BDR, according to the Australian Biodiversity Information Standard (ABIS), and the ecological data will be made available to end-users (DCCEEW 2024c). Together, these protocols, system and standards form EMSA. Such standardised environmental monitoring protocols and associated systems have not been used previously for federal government-funded NRM initiatives, so the new approach raises important questions about integration with NRM practices and socio-cultural norms for stakeholders across local, regional and national scales.

Box 1 Overview of EMSA (DCCEEW and TERN 2024) In summary, EMSA, the Ecological Monitoring System Australia, is a set of nationally standardised survey modules and data systems aimed at improving the collection, management and storage of ecological field data. This includes:

A manual detailing comprehensive instructions for a range of ecological field survey modules

A field data collection App called ‘Monitor’

 • A centralised data management and storage system - the Australian Government’s Biodiversity Data Repository

EMSA will:

 • Establish best practice monitoring protocols that support a range of project needs and outcomes

 • Improve understanding and measurement of the effectiveness of NRM actions

 • Strengthen the evidence used to assess the achievement of outcomes

Facilitate better access to and re-use of ecological survey data for adaptive management, research and policy

### Achieving Social Change for Successful Ecological Monitoring Standardisation

Although implementing an approach such as EMSA may be broadly viewed as worthwhile, previous research suggests that if there are significant socio-cultural difficulties with implementation, required changes in ecological monitoring will be unsuccessful (Biber 2013; Sanchirico et al. 2014; Michener 2015). The implementation of data standardisation is emerging as an important social issue in environmental management as policy and actions are required to response to ecological concerns at large scales. Any resulting social tensions in association with monitoring programmes can undermine environmental management success. Social conflict may be particularly difficult to resolve because scientists tend to focus on data and hypotheses, and can underestimate other values, such as socio-cultural significance or perceptions of uncertainty, key to the social processes of environmental monitoring and management (Sabatier and Jenkins-Smith 1993; Doremus 2007; Pritchard et al. 2022). In response, there are a range of methods emerging for overcoming social conflict during technical transitions and facilitating successful monitoring outcomes.

The importance of education for overcoming socio-cultural barriers to ecological monitoring standardisation is now well established. If stakeholders hold entrenched negative perceptions of a monitoring approach for reasons that lack strong evidence, education can fill gaps in understanding to facilitate adoption (Sharma and Monteiro 2016). NRM service providers have identified that they are open to adopting a standard method if they can see the value of the approach (Cox 2021). Indeed, previous research suggests that the better participants understand the objectives and value of a programme, the more closely they will follow procedures accurately (Gitzen et al. 2012; Biber 2013). For that reason, flexibility is allowed within EMSA to record information beyond data strictly required by the protocols, which will be important for maintaining participant interest and guiding evolution of the monitoring approach. It has been recommended that service providers be supported through education and stewardship programmes that demonstrate the intrinsic value of a standardised methodology (Cox 2021). In fact, as is discussed further below, part of the goal of this research is to inform training to accompany EMSA implementation.

Another method of overcoming socio-cultural challenges to effective monitoring is through the development of mutual understanding to enable collaboration among stakeholders (Allen et al. 2011; Biber 2013; Johannsson et al. 2022). Many successful long-term monitoring studies are built on partnerships between people with differing but complementary skills, including those concerned with the human dimensions of ecological monitoring, such as policy-makers (Danielsen et al. 2009; Lindenmayer et al. 2012; Ferrario et al. 2022). A transdisciplinary approach increases the relevance and impact of long-term ecological monitoring (Lynch et al. 2015). While improved communication between stakeholder groups may be sufficient for addressing low-level disagreements (Sanchirico et al., 2014), collaborative monitoring, where conscious information seeking is followed by shared critical analysis, has been found to promote learning and consensus building to inform NRM decisions (Cundill and Fabricius 2009). Increasingly, such collaboration is expanding to include professionals beyond science, whose work or interest involves the socio-cultural aspects of monitoring, such as social scientists, economists, artists and the general public (e.g. Robin et al. 2010; Sanchirico et al. 2014; Venus and Sauer 2022). Opportunities for scientists, managers, industrial and public stakeholders to meet and exchange experiences and expectations can facilitate multidisciplinary monitoring projects. By investigating stakeholders’ experiences of collaboration with DCCEEW and TERN during the development of EMSA, this research also initiates a social learning process of informing future opportunities for such collaboration.

### Aim of this Research

This research aims to gauge the perceptions of a preliminary sub-set of Australian ecological monitoring stakeholders in order to identify and begin to understand the socio-cultural challenges to EMSA implementation. How these challenges can be addressed will be key to ensuring successful implementation of the protocols, but will also be increasingly important for the effective integration of monitoring across scales and societies (Kühl et al. 2020). While this article identifies key stakeholder perceptions of and gaps in understanding related to standardised ecological monitoring, it also makes suggestions on how the approach could help to inform an emerging new era in environmental management in Australia.

## Methods

A qualitative approach was undertaken to review perceptions of the introduction of the EMSA protocols amongst NRM practitioners and other key stakeholders (Grypma et al. 2023). This research was conducted during July and August of 2023. Focus groups with TERN staff provided a valuable starting point to investigate the socio-technical context in which TERN operates. The discussions provided initial context and depth of understanding regarding stakeholders’ thoughts and experiences pertaining to EMSA (Ryan et al. 2014). In such a manner, the focus groups assisted in guiding the stakeholder interviews by providing key points of consideration for interviews. Together with the focus group data, the interviews highlight the wide range of perceptions that exist in relation to standardised ecological monitoring in Australia. Small scale, in-depth qualitative analyses, such as has been undertaken, here are powerful methods for drawing out key narratives on environmental management to critically evaluate approaches (Otto-Banaszak et al. 2011; Rosenbloom 2017; Bardsley et al. 2018). This study is unique in its examination of the perceptions of ecological monitoring standards and system developers, data collectors and end-users in conjunction (Cf. Chase and Levine 2018; Alblas and van Zeben 2023). The breadth of the discussion allows for comparison of the observations and opinions of these cohorts, enabling the development of a holistic picture of the socio-cultural processes of standardising ecological monitoring in Australia.

### TERN Focus Groups

Two preliminary focus groups were conducted with TERN staff involved in developing EMSA, to discern any socio-cultural challenges they anticipate or have encountered while engaging other stakeholders during the protocol development process. The first focus group involved five EMSA staff who were involved in a project support officer capacity, and the second involved two staff who were primarily involved in project management and communications. Each focus group ran for approximately one hour and twenty minutes.

Focus groups are regularly used to evaluate programme and policy implementation (Ryan et al. 2014). Asking stakeholders, through a focus group, to identify potential barriers to implementation before a programme has started is an effective method of rapidly informing actions at minimal cost (Gilmartin et al. 2019). Participants were emailed the list of discussion questions a week prior to their session and were invited to individually note their responses to the questions in their own time. The three pre-prepared questions most relevant to socio-cultural challenges to EMSA implementation then guided the group discussions. Notes taken during the focus group focussed on the participants’ verbal responses and prompted follow-up questions, while audio recorded and transcribed data ensured quotes were accurately captured.

### Semi-structured Interviews

Twenty semi-structured interviews were conducted with a diverse range of stakeholders who have, or will soon be, engaging with EMSA in their work across Australia. This included those involved in developing and managing EMSA, those who will be involved in collecting data through protocol implementation, and those involved in using the resultant data via the BDR. Specifically, participants included ecologists from both government agencies and academia, NRM staff involved in project design and reporting, and DCCEEW Nature Positive Integration division staff (See Table 1).

**Table 1:** Semi-structured interviews: EMSA stakeholders

Type of stakeholder	Number of interviews conducted
NRM region professional	10 consisting of:
	• 2 with Tasmanian regions,
	• 3 with South Australian regions,
	• 2 with New South Wales regions,
	• 1 with a Western Australian region,
	• 1 with a Victorian region, and
	• 1 with a Queensland region.
DCCEEW EMSA professional	5
NRM coordinator	2
End-user	2
Academic researcher	1

Interviews ranged in length from forty-five to ninety minutes and were conducted either face-to-face or remotely via Zoom. In the pursuit of anonymity, interviewees are referred to by general descriptors of their roles [See Table 1]. It was important to differentiate the participants in this manner in order to compare and distinguish responses by background. ‘NRM coordinator’ refers to a person who works as a conduit or facilitator of knowledge sharing between EMSA stakeholder groups such as NRM regions, state and commonwealth government agencies. ‘End-user’ refers to a representative of an organisation that will make use of the ecological monitoring data produced by EMSA. This method of participant anonymity was designed in accordance with the National Statement on Ethical Conduct in Human Research and approved by the University of Adelaide Human Research Ethics Committee (approval number H-2023-126).

#### Interview Participant Selection

Participant selection was not intended to be socially representative or comprehensive, but rather the approach aimed for in-depth conversations with a range of key stakeholders. The sample was stratified to obtain responses from a range of stakeholders across places and systems, and their answers are used to warrant the arguments on the opportunities and challenges of implementing EMSA. First, the researchers compiled a list of potential participants in association with the NRM Regions Australia Knowledge Broker to ensure a variety of stakeholders of different backgrounds and at different levels of their organisation were interviewed. The method is consistent with statistically non-representative stratified sampling, as variables were defined based on a hypothesis that those of different backgrounds will encounter or generate specific socio-cultural challenges to EMSA implementation (Trost 1986). Variables included stakeholder organisation (e.g. government or NRM service provider), region of Australia working within, and nature of engagement with EMSA (e.g. as a developer, data collector or end-user) (Table 1).

### Thematic Analysis

To ascertain and interpret key perceptions and socio-cultural challenges to EMSA implementation based on the focus groups and interviews conducted, the data was analysed using techniques of thematic analysis, as set out by Braun and Clarke (2021). Following transcription, the focus group data, including individual participant written responses, and each interview was read through and initial analytic insights noted. This familiarised the researchers with the entire dataset. Subsequently, the process of generating and elaborating concepts was commenced using the interpretative, inductive techniques of coding. Coding was both semantic (capturing the explicit or surface meaning) and latent (capturing more conceptual or implicit meaning) (Braun and Clarke 2021).

Consistency and accuracy of coding was achieved, and the intrusion of bias limited, through application of the techniques of thorough repetitive reading, constant comparison and questioning by the first two authors (Charmaz 2006; Corbin and Strauss 2015; Creswell 2014; Glaser and Strauss 1967). Constant comparison involves examining data both within and between documents, in this case the interview transcripts, to group together data that are conceptually similar. Questioning involves asking various questions of the data in order to draw meaning from them. Typical questions included, ‘What are the issues, problems, and concerns here?’, ‘Who are the actors involved?’ and ‘How do they define the situation?’. The coding process, allowed for the identification of repletion in key concepts that were integrated where appropriate and grouped into broader themes, or narratives, to highlight important messages (Moskwa et al. 2018). We summarise the key findings from the qualitative data using tables developed from a quantification of repeated narratives, and elaborate on those messages with evidence warranted from the qualitative data following the approach taken by Bardsley et al. (2023).

## Results

The thematic analysis of the focus groups and interviews revealed a range of perceived potential advantages of EMSA (Table 2); however, we also discovered a range of perceived socio-cultural challenges relating to EMSA and its implementation (Table 3). There was a clear validation across the respondent cohort of an attempt to develop a national approach to ecological monitoring: to be able to warrant national decisions; to enable continental-scale research; to more accurately guide national reporting; and to generate high quality, reproducible datasets (Table 2). These results are important because they strongly support the attempt to develop EMSA. Nevertheless, multiple key concerns were raised at the approach taken. Some respondents were concerned with retention of regional autonomy and the potential risk of centralisation of monitoring within government (Table 3). Yet, for the majority, such a loss of independence would be tolerable given the potential benefits of systematic monitoring, if the approach could be made adaptable to local situations, could be enabled by co-design and could be effectively resourced. We detail several of these key challenges further below.

**Table 2:** Key advantages of EMSA perceived by stakeholders

Advantage of EMSA mentioned	Respondent																					
	NRM region professionals										DCCEEW EMSA professionals					NRM knowledge brokers		End-users		Academic	TERN focus groups	Total
	1	2	3	4	5	6	7	8	9	10	11	12	13	14	15	16	17	18	19	20	21	
Enable/validate national ecological research	x	x			x	x	x	x	x	x	x	x		x	x	x	x	x		x	x	17/21
High quality national level information	x			x	x	x	x	x		x	x	x	x	x	x	x	x	x	x	x		17/21
Accountability for public funding	x	x			x	x			x		x	x	x		x	x	x	x	x		x	14/21
Reliable collection and storage of data			x	x	x			x	x		x	x	x	x		x	x		x		x	13/21
Guide reporting (e.g. SOE, international)	x						x				x	x	x	x		x	x	x	x	x	x	12/21
Enable reproducible science-based monitoring		x	x		x		x				x			x	x	x		x	x		x	11/21
Enable longevity of ecological monitoring	x					x			x			x	x		x	x			x		x	9/21
Easily accessible data							x		x		x	x				x		x	x		x	8/21
Flexible systems that adapt to needs									x		x		x	x	x	x	x				x	8/21
Create efficiencies in data collection/ management/ use			x						x		x	x	x			x			x		x	8/21
Normalisation of national-scale monitoring						x						x	x			x			x	x		6/21
Enable investment in ecological outcomes					x						x		x	x		x		x				6/21

**Table 3:** Key socio-cultural challenges of EMSA perceived by stakeholders

Sociocultural challenge mentioned	Respondent																					
	NRM region professionals										DCCEEW EMSA professionals					NRM knowledge brokers		End-users		Academic	TERN focus groups	Total
	1	2	3	4	5	6	7	8	9	10	11	12	13	14	15	16	17	18	19	20	21	
Insufficient capacity to implement protocols	x	x	x	x	x	x	x	x	x	x	x	x	x	x	x	x	x	x	x	x	x	21/21
Data utility and access for regional projects	x	x	x	x	x	x	x	x	x	x	x	x	x	x		x	x	x			x	18/21
Adaptability of EMSA protocols/tools/system	x	x	x	x	x	x	x	x	x	x	x	x		x	x		x	x	x		x	18/21
Communications regarding EMSA roll-out	x	x		x	x	x	x	x					x	x	x	x	x	x	x		x	15/21
Attachment to current practices		x	x	x		x		x		x	x	x	x	x	x	x			x	x	x	15/21
Demonstration of clear EMSA purpose/value	x	x				x	x	x		x		x	x	x			x	x	x	x		13/21
Giving and responding to feedback	x	x	x	x	x		x	x	x			x	x			x					x	12/21
Weaken local relationships and knowledge		x	x	x	x	x			x	x						x	x	x		x		11/21
Need to see short-term advantages	x	x	x				x			x	x	x	x	x		x	x					11/21
Commonwealth/State government coordination		x	x				x		x		x				x	x	x		x		x	10/21
Initial EMSA development consultation		x	x			x	x	x	x	x		x				x					x	10/21
Autonomy of NRM regions		x	x		x		x			x						x		x				7/21

### Insufficient Capacity to Carry Out EMSA Protocols

Respondents noted that natural resource management, and ecological monitoring in particular, is not well resourced. All respondents raised the issue of adequate financial and labour resources being required to facilitate the transition to systematic monitoring, especially in regional and remote areas. Some highlighted that the potential for a lack of financial support was their primary concern regarding EMSA protocol implementation, for example:

Our biggest concern is that we can’t implement [the EMSA protocols] because we’re not resourced to implement them. They will add considerably to the cost of our operations, and if that [cost] has to come out of projects, we might say “We won’t achieve these outcomes on the ground, and [instead] we’ll spend [funds] on monitoring the limited outcomes that we’ve achieved.” (NRM region professional 4)

Part of that concern originates from the recent shift in the way NRM activities are funded by the Commonwealth Government from a grant-based funding model to a procurement model, which largely assigns NRM regions with the role of a service provider (Rolfe et al. 2017). Respondents noted that this method of funding gives the Commonwealth more power to demand particular outcomes, which regions may struggle to apply. Stakeholders were concerned that EMSA implementation would generate additional bureaucratic burdens limiting time to develop understanding of local issues or implementing appropriate management interventions. The following quotes show stakeholder’s experience of NRM funding cycles running over short time periods and so not sufficiently covering many key management operations over the longer term.

The realistic impact that you can have in five years with the dollars provided is sometimes quite limited. Sometimes partnerships and engagement are the main outcome… For example, you’ve managed to get a memorandum of understanding with a traditional owner group… [in that case] you’re not paying for an environmental outcome, and you need to be clear, “In the next five years, we’re just scratching the surface of trying to fix this problem.” The TERN protocols are [irrelevant] in that situation. (NRM region professional 3)

How difficult is it to establish relationships with businesses, land councils and enterprises in remote regions, from Canberra, over a 4 to 5 year period? And then pull funding, or know funding is going to end, or maybe keep going into the next programme… (DCCEEW EMSA professional 15)

### Data Utility and Access for Regional Projects

Data is used all the time by local and regional environmental management groups to review outcomes of environmental management interventions and make decisions for future actions. A widespread concern emphasised by NRM region professionals was that data gathered utilising the EMSA protocols and associated tools would be insufficient to reveal whether their local projects, including Commonwealth funded activities, are producing desired outcomes. NRM Coordinator 17 summarised this perception and articulated the associated socio-cultural issue well:

What the Commonwealth tends to revert to is measuring biodiversity the same way everywhere, at a national level. You get this complete incongruence with what they’re investing in, which is very local, and what they’re measuring… that means they run into problems. I think there’s also a social element: you’re engaging regional boards, staff and communities in a process which is very local to them, and then you’re asking them to engage in this evaluation process, which has nothing to do with them. Essentially, you’re asking them to go out and measure things which, from their perspective, have no bearing on whether they’re being effective or not. (NRM Coordinator 17)

While the protocols are organised to provide baseline data that could answer a range of ecological questions, there was a perception that such scientific analysis was seen as being difficult to implement at regional scales. In fact, several respondents argued that the best local outcomes for them came when high quality monitoring could be undertaken locally with limited external “interference”. Another widespread concern exemplified in the below quote was that EMSA data would not be readily available in a form that they could use to easily make decisions or verify actions.

We haven’t been given any indication how we get our data back. We know we have to enter data into the app, and it goes into the Biodiversity Data Repository, but there is no indication of how we get it back, and no indication of what we do with it then, because if you have one plot with thousands of individual data points in it; that’s beyond the capacity of a lot of NRMs to analyse. (NRM region professional 2)

### Adaptability of EMSA Protocols

Over 85% of participants emphasised that the EMSA protocols need to be made adaptable to suit unique ecological circumstances, with the capacity to evolve as implementation commences and technical or logistical issues are identified. TERN staff recognised current inflexibility in plot size as well as a probable need for further adaptation as the protocols begin to be used:^1^

I think [the protocols] are pretty flexible, but plot size will be a primary limiting factor, and [ensuring the protocols can be used with] different plot sizes is something that we’re going to explore. There will be heaps of situations where you need to adjust plot size, and at the moment there isn’t flexibility to do that, but a lot of the protocols are fairly broad. They have been designed to be applicable to different systems. There may be a period of figuring out how things need to be changed or adapted to capture everything. (TERN focus groups)

### Opportunities to give Feedback and Receive Responses

In a third set of questions, we asked respondents to elaborate on approaches they were suggesting to support EMSA implementation. Most responses linked to the most important issues highlighted above, with ideas for either better exploitation of opportunities from systematic ecological monitoring, or avoidance of potential negative impacts or constraints. Clear communication, and in particular opportunities to give feedback that is subsequently acknowledged and responded to, was the key theme of respondents’ suggestions for improvement. Other themes that we do not have capacity to present here were: the need for learning through the transition to using EMSA, accessible technical support, multimodal training, and support for indigenous monitoring aligned with the EMSA protocols. Many of those requests will evolve in the next phase of EMSA development in the form of help desks, videos, training and other interactions with stakeholders.

About half of respondents acknowledged giving and responding to feedback as an important social process to engage in during EMSA implementation. Even though there were some strong attempts at stakeholder engagement with a range of opportunities available to regional stakeholders, they felt there was insufficient acknowledgement or incorporation of that feedback into co-design of the protocols or monitoring approach (see the quote below, for example). This may have negatively influenced perceptions of EMSA early on and made some regional NRM professionals sceptical of the worth of further engagement with the process.

It didn’t feel like genuine consultation. There was no in-depth consultation, particularly on what NRM needs are, and how it would actually work… There was a spreadsheet sent out for feedback. I asked nine questions, and nothing happened… I chased it up: “I haven’t gotten any answers.” I’ve since received answers to four of my questions… This [interview] is the first proper opportunity I’ve had to provide any feedback to the department. (NRM region professional 2)

There is a communication challenge to explain complex scientific decisions that aim to meet universal goals on a general scale that will respond to all regional concerns. For example, TERN staff explained how some feedback ideas on draft protocols could not be appropriately integrated into the approach in the short-term, but this might not have been effectively communicated to regional groups:

Throughout the whole project, we’ve repeatedly told [NRM service providers] what we’re doing. We’ve asked them for feedback. We’ve gotten very little feedback. (TERN focus groups)

Therefore, a key narrative was that a major transition in a scientific approach would benefit from regular communication and greater direct participation from key stakeholders to ensure changes are understood and to facilitate ownership of new technical processes.^2^ In fact, several stakeholders argued that NRM service providers should have been far more directly involved in the initial phase of crafting a solution to the problem of inadequate ecological monitoring, for instance:

I don’t feel the NRM sector was at all involved in the discussion of how we can make things better. Before going to TERN and saying “Solve this problem for us”, have this conversation with the regions. They’ve got us out there in the regions that they’re contracting projects with, we know better than anyone about the implementation side, and I feel like we weren’t consulted at all. (NRM region professional 6)

Authentic engagement with NRM stakeholders for co-design will be important as EMSA implementation continues. One idea suggested by numerous respondents was the importance of multimodal training in EMSA to assist in building relationships and confidence in the new system. NRM region professional 4 expressed an opinion that thorough training will be needed, based on his previous experience implementing monitoring standards:

When the [state-based] vegetation benchmarks project was rolled out…we went through a 2.5-year training process - the focus was on training left, right and centre to target as many people as possible—and we’re still trying to encourage the uptake of that methodology. (NRM region professional 4)

Some volunteer groups, such as citizen scientists, may be more reluctant to apply the protocols and use the app and related systems. Some regions or groups with less capacity or unique monitoring needs may need targeted direct training and on-going assistance to effectively integrate the protocols into their monitoring systems. One suggestion was for further face-to-face engagement with NRM sub-regional partners, who in turn could become change-leaders within their region:

I think having champions associated with [EMSA] is really important. There are a lot of events that happen in the bush, where you can just book an hour to be face-to-face with people. There’s nothing better. Once you get key people on side, then the ideas will spread and be taken up. (Academic)

Although it was beyond the scope of the initial phase of EMSA development, the co-design of regional projects was also seen as a highly valuable tool for successful implementation in the future, as illustrated in the below quote. The negotiation of projects in association with monitoring, and the choice of application of particular protocols suited to local needs would be a vital step in the acceptance of the new monitoring approach.

I’d like to see more intensive engagement with us as delivery partners for this type of work. We [could] assess the protocols and their applicability [together with DCCEEW]. I hope there’s a fair bit of opportunity to opt-out where we can justify why we’re not following those protocols. Because some of the work we do is simply community engagement. It’s not about collecting rigorous monitoring data. (NRM region professional 5)

## Discussion

This study demonstrates how an array of complex social, institutional, and ecological issues, operating at various scales, can emerge from a change in scientific approach within a community of practice. Acknowledgement of the diversity of experiences of monitoring standardisation within the NRM community is a first step in resolving that complexity. Complex systems require a multivariate, integrated approach to environmental management (Bellamy et al. 2001; Cundill and Fabricius 2009), and governance approaches like adaptive co-management have come about as a means of dealing with such complexity and uncertainty (Ollson et al. 2004). These approaches rely on collaboration among a diverse set of actors, whereby actions are coordinated by stakeholders in a self-organising and self-enforcing manner. Throughout our research, stakeholders highlighted the need to retain such autonomy and flexibility throughout our research, so their involvement in how the protocols evolve could enable NRM practitioners to coordinate associated actions and self-organise to facilitate appropriate regional refinement of the methods for monitoring standardisation. To avoid the weakening of regional cooperation and community networks, local relationships and knowledges will need to be acknowledged as unique and highly valuable. Allowing for some variation that responds to that socio-ecological complexity will be important, with targeted exemption from protocols and revisions, at least for a period during initial EMSA implementation. This is consistent with the practise of holistic ecological monitoring, which aims to advance social inclusiveness through evolutionary change (Ferrario et al. 2022). This process has already begun and significant progress has been made since our focus groups and interviews were conducted in July and August of 2023 (see Box 2).

Our findings indicate that social reflexivity will be a vital tool to guide learning to address the complexity involved in standardisation of ecological monitoring. New approaches to environmental management will have to account for the specific attributes of socio-ecological systems in order to overcome the unprecedented challenges that will arise in the wake of future global change. While both informal and institutionalised reflexivity require allocation of limited time and resources, when intentionally undertaken, reflexive processes can be integrated into adaptive management at multiple points, helping environmental management stakeholders learn how to better reach their goals (Mol 1996; Pearson and Bardsley 2022; Pienkowski et al. 2023). Reflexivity helps to play a transformative role, supporting practitioners to regularly re-evaluate their goals and methods to meet changing needs and overcome constraints. Although the term was not mentioned explicitly by respondents, they expressed a deep understanding of and desire for reflexive approaches to monitoring standardisation. The need for greater reflexivity is not limited to ecological monitoring approaches in Australia. Environmental management is increasingly embracing the explicit use of social learning processes to transform governance and practice, with work by McLoughlin et al. (2020), Boyce et al. (2022), and Gale (2006), articulating how formal processes of social learning for improvements in water management in the Murray-Darling Basin, values-based conservation, and cleaner production initiatives for corporate sustainability, respectively.

EMSA itself was formed from a reflexive process, as an outcome of a comprehensive re-evaluation of Australian ecological monitoring and reporting, and is being reformed through contemporary stakeholder feedback (Box 2). Since the surveys were conducted, a number of these recommendations have been adapted and undertaken by both DCCEEW and TERN resulting in a great number of the concerns being addressed. Our results, and the actions of this social research in itself, is showing that the need for standardisation was broadly endorsed as a response to the shortfalls of current monitoring practice. Education about the difficulties of a high-level transformation to a procurement funding model within NRM, and the advantages and disadvantages of that model is required to ensure regions understand the context within which DCCEEW and TERN stakeholders are operating. Beyond the need for awareness-raising, the major concerns of protocol adaptability, consequent data utility, lack of consultation and adequate opportunities to give feedback conveyed by NRM stakeholders reflect how a perceived lack of allowance for unique local attributes and needs hindered initial EMSA implementation.

The lack of resourcing of Australian NRM in general, and concerns with insufficient extra support for this ‘upgrade’ of ecological monitoring in Australia, emerged as the key narrative. Importantly, our results show that capacity limitations are not only about funding, but also relate to an NRM organisation’s ability to take the time to engage in, and benefit from, collaboration and learning activities. Aside from providing more funding, making social learning an explicit objective of the change process is necessary to build social capacities (Fernández-Giménez et al. 2019; Dimmock et al. 2014; LoSchiavo et al. 2013). Therefore, resourcing for EMSA implementation needs to not only update tools and technical activities, but also facilitate learning processes to generate the social licence for the new monitoring system. Our results support this conclusion, with respondents calling for multimodal training, the development of local champions to spearhead implementation, and co-design of projects utilising the protocols.

The standardization of the EMSA is generating new challenges for co-design of monitoring to guide local environmental management. To ensure that stakeholder concerns do not lead to major conflicts there will need to be ongoing improvements in the communication between researchers and applied sectors to enhance reflexive collaboration for adaptive environmental management (Brownson and Fowler 2020; Allan and Watts 2018; Hagell and Ribic 2014). Practically, authentic ongoing engagement between TERN staff, government and state and regional-level stakeholders will be vital and a community-of-practice approach is designed to generate networks for discussion to reinforce that people’s suggestions were being, and will continue to be taken into account (Box 2). Respondents’ concerns about a perceived lack of genuine consultation and acknowledgement of feedback, reveal the importance of clear communication at all stages of an environmental management project that aims to standardise and streamline practices (Månsson et al. 2023). When researchers work closely with science end-users, as we have attempted to model through this research, constructive change is enabled because collaboration can immediately and directly identify and work to address social obstacles. Trust building, dialogue and active involvement have been identified as among the most recognised qualities of collaboration (Fiest et al. 2020). In modelling an approach to develop understanding of the socio-cultural challenges to the implementation of a new environmental management approach, our research demonstrates the advantages of such transdisciplinary learning to inform the standardisation of ecological monitoring.

Box 2 Improvements Currently being made to EMSA Context: Interviews that informed perceptions of EMSA for this study occurred greater than 12 months ago, at a time when the EMSA protocols were being developed and the NRM community were being made aware of the change in process for both ecological monitoring, but also their procurement model for NRM services. Since the interviews, the EMSA system has been iteratively refined with a release of finalised protocols released in March 2024 and the app in May 2024.

Activities to address stakeholder concerns:

Release of the EMSA website (emsa.tern.org.au), including:

 • Explanation and instruction videos (field and app usage)

 • Development of ‘how to’ guides

 • The creation and resourcing of two help desks

  ○ One focused on ecological protocols and usage

  ○ One focused on technical support for the app, devices for use, etc.

 • A document library, including the most recent versions of all protocols, decision support tools for protocol selection and several case studies.

 • Details on planned field training courses (a week-long field training course is currently being developed)

 • Links to previous webinars explaining the system

 • A community forum (for peer-to-peer learning – soon to be released)

Creation of an EMSA Community of Practice (CoP) - This has met every 6 weeks during 2024 and includes the occasional formal presentation, along with a community discussion to ask questions and provide feedback on both the protocols and the data collection app. When specific foci are requested then a session is run specifically on that topic with the aim of a general CoP interleaved with a specific topic CoP (e.g. how to get started with the app).

In addition, DCCEEW run a structured exemption process. Stakeholders who would like to apply for an exemption from using specific EMSA protocols or tools fill out a form that collects information on their project aims, why they think the EMSA protocols are not appropriate, detail on whether there is a more appropriate method and why, and detail on how they will make the resulting data findable, accessible, interoperable and reusable (FORCE11, 2021). This application is then assessed by the EMSA protocol advisory group (PAG) who then assess the validity of the exemption and either approve or deny the application for exemption. Often a compromise is found where some elements of EMSA can be incorporated along with more localised methods.

## Conclusion

According to our research, environmental management stakeholders are recognising multiple values of the standardisation of ecological monitoring. However, as a unifying approach to monitoring data is applied, refinement of approaches to suit local contextual situations and desired outcomes are crucial to successful implementation. Most of the commonly mentioned advantages to standardisation are most obvious at national or scientific scales rather than providing opportunities to improve local or regional environmental knowledge and management. To generate local benefits, local people must be integrated into the design and application of the new monitoring approach. Reflexivity is already starting to be included into EMSA implementation through opportunities for project co-design and a protocol exemption process, but there are also key social processes beyond such mechanisms related to the transition that will need to be developed in the future. Further research is needed to investigate appropriate methodologies to develop and implement the approach to ecological monitoring standardisation our study supports.

There are both important academic and practical values in undertaking research to analyse how transitions in ecological monitoring will be accepted by scientific and NRM communities. Social knowledge on how best to implement technological and procedural change will be vital to inform a new era in environmental management: an era that will increasingly incorporate environmental change; green investment; regeneration of multifunctional landscapes; increasing natural hazard risk; and a broad range of new relationships between people and ecology. In this case, the normalisation of high-quality national level ecological monitoring to achieve an improvement in the understanding of the national environment, and increased accountability of funding for public investment were the most widespread perceived advantages. The most commonly perceived socio-cultural challenges centred around the adaptability of EMSA methodologies for NRM service providers, and the need for adequate resourcing and prioritisation of relationships with stakeholders. To respond to such socio-ecological complexity, strong participatory approaches will be needed to develop effective technical solutions – and these changes in the EMSA approach are already starting to be implemented. Such processes of mutual social learning between scientists and practitioners that can enable ownership of the new approach and lead to co-design of policy and practice must be normalised to guide effective transitions in environmental management.

## Footnotes

1 Greater flexibility in plot size has now been funded to be implemented in the next phase of the EMSA programme (see Box 2).

2 TERN is now implementing conferences of the parties every 6 weeks to respond to this concern (see Box 2).
